# MicroRNA-19b Downregulates Gap Junction Protein Alpha1 and Synergizes with MicroRNA-1 in Viral Myocarditis

**DOI:** 10.3390/ijms17050741

**Published:** 2016-05-18

**Authors:** Junyi Lin, Aimin Xue, Liliang Li, Beixu Li, Yuhua Li, Yiwen Shen, Ning Sun, Ruizhen Chen, Hongfei Xu, Ziqin Zhao

**Affiliations:** 1 Department of Forensic Medicine, School of Basic Medical Sciences, Fudan University, Shanghai 200032, China; 13111010032@fudan.edu.cn (J.L.); amxue@fudan.edu.cn (A.X.); 11111010016@fudan.edu.cn (L.L.); libeixu@fudan.edu.cn (B.L.); 12111010086@fudan.edu.cn (Y.L.); shenyiwen@fudan.edu.cn (Y.S.); 2 Department of Pathophysiology, School of Basic Medical Sciences, Fudan University, Shanghai 200032, China; sunning@fudan.edu.cn; 3 Key Laboratory of Viral Heart Diseases, Ministry of Public Health, Shanghai Institute of Cardiovascular Diseases, Zhongshan Hospital, Fudan University, Shanghai 200032, China; chenruizhenzs@gmail.com; 4 Department of Forensic Medicine, Soochow University, Suzhou 215123, China

**Keywords:** viral myocarditis, microRNA, gap junction, miR-19b, miR-1

## Abstract

Viral myocarditis (VMC) is a life-threatening disease that leads to heart failure or cardiac arrhythmia. A large number of researches have revealed that mircroRNAs (miRNAs) participate in the pathological processes of VMC. We previously reported that miR-1 repressed the expression of gap junction protein α1 (GJA1) in VMC. In this study, miR-19b was found to be significantly upregulated using the microarray analysis in a mouse model of VMC, and overexpression of miR-19b led to irregular beating pattern in human cardiomyocytes derived from the induced pluripotent stem cells (hiPSCs-CMs). The upregulation of miR-19b was associated with decreased GJA1 *in vivo*. Furthermore, a miR-19b inhibitor increased, while its mimics suppressed the expression of GJA1 in HL-1 cells. When GJA1 was overexpressed, the miR-19b mimics-mediated irregular beating was reversed in hiPSCs-CMs. In addition, the effect of miR-19b on GJA1 was enhanced by miR-1 in a dose-dependent manner. These data suggest miR-19b contributes to irregular beating through regulation of GJA1 by cooperating with miR-1. Based on the present and our previous studies, it could be indicated that miR-19b and miR-1 might be critically involved in cardiac arrhythmia associated with VMC.

## 1. Introduction

Viral myocarditis (VMC) is a severe cardiovascular disease characterized by inflammatory infiltration in the myocardium. It is one of the most challenging problems in cardiology, which can lead to heart failure or cardiac arrhythmia and contribute to sudden unexpected death in young and healthy individuals. According to statistics, VMC accounts for up to 12% of sudden deaths in patients under 40 years of age and becomes a leading cause of dilated cardiomyopathy which may result in deaths in 50% of patients 1–2 years after diagnosis [1,2,3,4]. Coxsackievirus B_3_ (CVB_3_) is the most common etiology that usually causes cardiac arrhythmia and sudden cardiac arrest [5]. Moreover, the therapeutic interventions of CVB_3_-induced myocarditis remain limited and nonspecific [1]. The exact mechanisms of how CVB_3_-induced myocarditis are also not well understood.

Gap junctions are intercellular constitutes that mediate electrical coupling between cardiac myocytes in the heart [6]. Gap junction protein α1 (GJA1) is the major gap junction protein in the heart and widely expressed in atrial and ventricular myocytes [7]. It is primarily responsible for the electrical synchrony of cardiomyocytes. Dysfunction or disorganization of GJA1 can increase the propensity for arrhythmias [8]. Reduction of GJA1, particularly in VMC, has been implicated to decrease the conduction velocity and increase the arrhythmia susceptibility [9]. Hence, dysregulation of gap junction proteins is associated with arrhythmias.

Despite the strong connection between gap junctions and arrhythmias, regulation of gap junction proteins remains largely unclear until the discoveries of microRNA [10]. MicroRNAs (miRNAs) are a class of endogenous small noncoding RNA that regulate gene expression. They are generally regarded as negative regulators of gene expression via complementary base pairing to the 3’-untranslated region (3’-UTR) of their target mRNA [11]. In recent years, miRNAs have been implicated in a wide spectrum of pathophysiologic processes [12], including the disease of VMC. For example, the upregulation of miR-221/222 cluster correlates with the antiviral and inflammatory immune response to viral infection in VMC [13].

A previous study showed that miR-1 regulated cardiac arrhythmogenic potential by targeting GJA1 and KCNJ2 [10]. Our previous work also showed that GJA1 could be post-transcriptionally regulated by miR-1 in VMC [14]. In this study, we aimed to find out other miRNAs that were aberrantly expressed in VMC by microarray analysis. Our data identified miR-19b as a critical miRNA which was significantly upregulated and could synergize with miR-1 to control the expression of GJA1 protein in the development of VMC.

## 2. Results

### 2.1. CVB_3_ Infection Triggered Significant Myocardial Lesions

**Figure 1:**
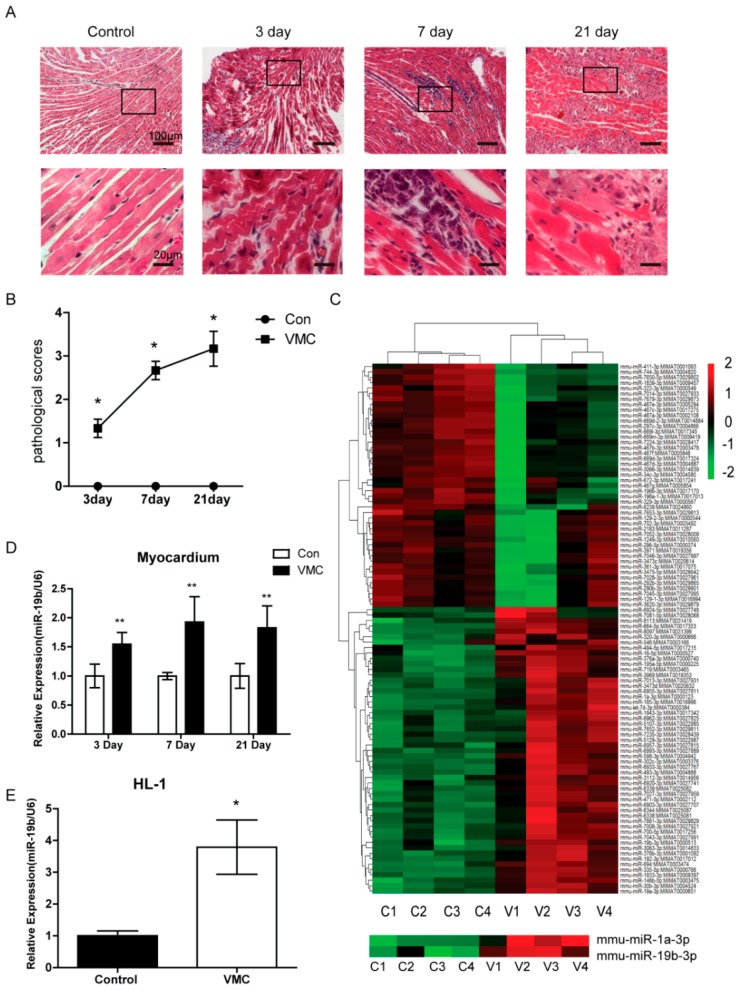
miR-19b was upregulated in the CVB_3_-induced viral myocarditis. (**A**) Histopathologic analysis of myocardium in VMC group and mock-infected group. Myocardium was normal in the control mice, while different extents of infiltrates were observed on day 3, 7 and 21. The bottom images are magnifications of upper ones. The scale bar of upper pane is 100 μm, and of the bottom pane is 20 μm; (**B**) The pathological scores were shown in different groups; (**C**) Heat map and hierarchical clustering of miRNAs. miR-19b and miR-1 were shown to be increased in VMC; (**D**) qRT-PCR analysis confirmed the miRNA microarray findings. All miRNA expression values were normalized against the U6 endogenous control. *n* = 6 for each independent sample; (**E**) qRT-PCR revealed miR-19b was increased in HL-1 cells infected CVB_3_, *n* = 3 per group * *p* < 0.05 and ** *p* < 0.01 *vs*. control.

The CVB_3_-inoculated mice developed severe symptoms of viral myocarditis, exhibiting weakness, lethargy, irritability and anorexia two days after inoculation. Histopathological observations of the cardiac tissues showed myocardium was mildly infiltrated with focal inflammatory cells on day 3. On day 7 after CVB_3_ infection, inflammatory infiltrates were diffused around the myocardium and in the myocardial interstitium. By day 21, inflammation was even severe with remarkable myocardial necrosis and fibrosis. In contrast, the control group showed no inflammatory cell infiltration, necrotic or fibrotic lesions in the hearts (Figure 1A). Accordingly, the cardiac pathological scores of mice bearing VMC significantly increased compared with the control ones (Figure 1B). These results suggested that CVB_3_ infection triggered significant myocardial lesions, and therefore confirming the successful establishment of VMC model in mice.

### 2.2. miR-19b Was Upregulated in VMC Model in Vivo and in Vitro

To screen which miRNAs were altered in the CVB_3_-induced VMC, we performed a microRNA microarray analysis of the heart tissues on day 7. It was shown that multiple miRNAs were altered (Figure 1C). We further narrowed down our targets to include only those miRNAs which were specifically expressed in the heart. It turned out that miR-19b was significantly upregulated in the VMC heart. Since miR-19b played a crucial role in the development of heart and was involved in cardiac arrhythmias [15], we selected it for our subsequent confirmative assays. Using the real-time PCR analysis, it was verified that miR-19b was increasingly upregulated in the heart throughout the progression of VMC, and peaked on day 7 compared with the control group (Figure 1D). To confirm miR-19b was upregulated in cardiomyocytes during VMC, we used CVB_3_ to infect the HL-1 cells which are immortalized mouse cardiac cells. The *in vitro* data also verified that miR-19b was increased in the cell model of VMC (Figure 1E). The results indicated miR-19b may play an important role in murine VMC model.

### 2.3. Overexpression of miR-19b Resulted in Irregular Beating Patterns in hiPSCs-CMs

**Figure 2:**
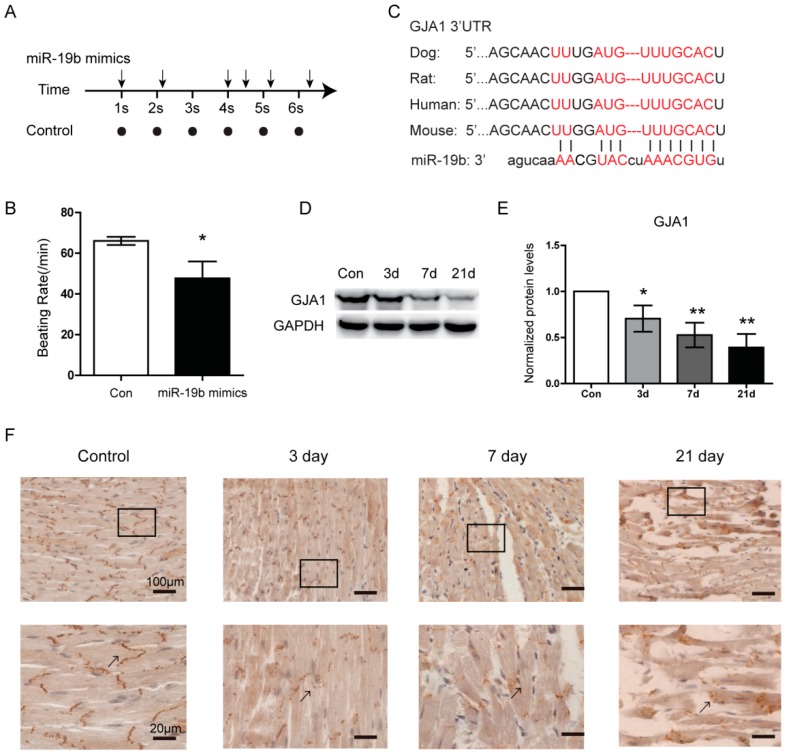
The expression of GJA1 was reduced in the heart of mice bearing VMC. (**A**) The representative beating pattern of cardiomyocytes treated with miR-19b mimics. Each arrow represented one beating of cardiomyocytes and horizontal axis showed the time intervals between two close beats. And the plots represented the control; (**B**) The beating rate of hiPSCs-CMs was shown in different groups; (**C**) The computational alignment of a potential conserved target site of miR-19b in different species. Red sequences represent the complementary bases between miR-19b and GJA1 3’-UTR in species including *Homo sapiens*, rat, dog and mouse; (**D**,**E**) GJA1 protein levels were detected at the indicated time of VMC mice model by western blot and the intensity quantification analysis. GJA1 was reduced as the development of VMC; (**F**) IHC analysis showed that GJA1 protein was reduced in VMC mice compared to normal mice. Black arrows showed the gap junctions became uncontinuous and represented that GJA1 was less expressed. Bottom images were magnifications of upper ones. Scare bars for upper panes are 100 μm and for bottom ones are 20 μm. *n* = 3 per group, * *p* < 0.05, ** *p* < 0.01 *vs*. control.

The human induced pluripotent stem cells derived cardiomyocytes (hiPSCs-CMs) have been proposed as a good model for cardiac arrhythmias [16,17]. To explore the effects of miR-19b upregulation on cardiac rhythm in VMC, we transfected miR-19b mimics into the hiPSCs-CMs. Our results showed that when miR-19b was upregulated, hiPSCs-CMs beat irregularly as manifested by an erratic rhythm (Figure 2A and Supplementary video 1, video 2), and the beating rate was significantly decreased as compared with control cells (Figure 2B). This result suggested that the enhanced expression of miR-19b could cause irregular beating patterns in hiPSCs-CMs and might explain the arrhythmogenesis in VMC.

### 2.4. GJA1 Was Predicted to Be Targeted by miR-19b and Was Downregulated in VMC

In order to explore the underlying molecular mechanism of miR-19b in VMC, we searched for potential regulatory targets of miR-19b using several bioinformatics methods, including TargetScan, miRWalk and miRanda. All three algorithms consistently predicted GJA1 as a target of miR-19b, whose putative target sequence is located in 1373–1386nt of the 3’-UTR of GJA1 mRNA (Figure 2C). To verify the possible regulation of GJA1 by miRNA-19b, we detected the expression of GJA1 in the heart from VMC models. Western blot assay revealed that the protein level of GJA1 was decreasing over the infection time (Figure 2D,E). Immunohistochemistry also showed that with the duration of VMC, GJA1 was less expressed between the myocardium. Gap junctions were clear and continuous in control hearts. However, they were irregularly and mildly stained 3 days after infection, and became uncontinuous or even missing with the progression of VMC (Figure 2F). These results indicated that GJA1 was decreased along with VMC progression and might be a result of the upregulation of miR-19b.

### 2.5. miR-19b Directly Regulated the Expression of GJA1 in Vitro

**Figure 3:**
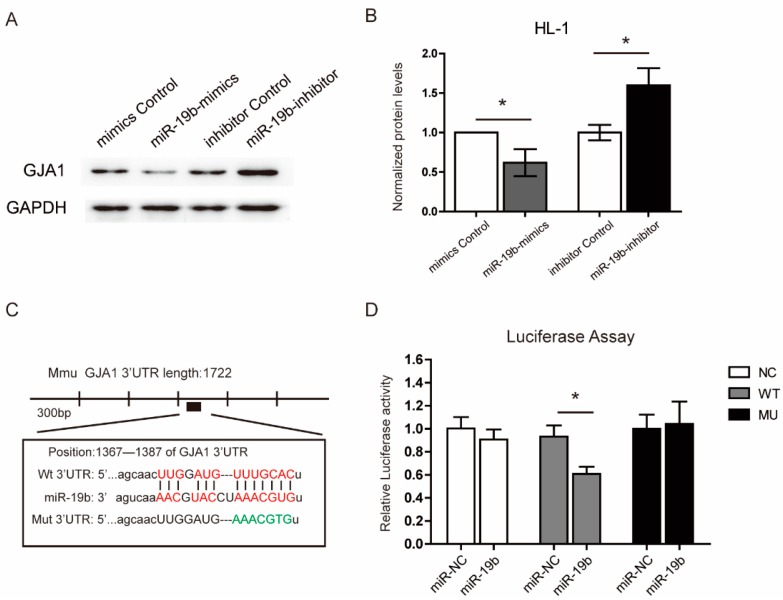
miR-19b directly targeted the 3’-UTR of GJA1 mRNA. (**A**,**B**) HL-1 cells were transfected with miR-19b mimics, inhibitor, or negative control respectively. Western blot assay revealed that the levels of GJA1 protein were negatively correlated with miR-19b; (**C**) Schematic representation of wild type and mutant binding sites of miR-19b in the 3′-UTR of GJA1. Red sequences represent the complementary bases between miR-19b and GJA1 3’-UTR in wild type, green sequences represent the mutant miR-19b binding sites in GJA1 3’-UTR; (**D**) Luciferase assay showed that the miR-19b transfection leads to decreased GJA1-3’-UTR-wt fluorescence intensity in comparison to the negative control (miR-NC), while the GJA1-3’-UTR-mut group or vector group showed no differences between the miR-NC and miR-19b-transfected groups. *n* = 3 per group. * *p* <  0.05 *vs*. miR-NC group (NC: negative control).

Next, the expression of GJA1 was detected in response to miR-19b treatments in mouse cardiomyocytes HL-1 cells. As shown in Figure 3A,B, GJA1 was dramatically suppressed by miR-19b mimics. In contrary, utilizing an established miR-19b inhibitor, we found that the inhibition of miR-19b significantly enhanced the expression of GJA1. As we have shown above, GJA1 was inhibited by miR-19b presumably by binding to the 1373–1386 nt of the GJA1 3’-UTR, therefore, we further verified the observation using luciferase reporter assay. The GJA1 mRNA containing the putative binding site of miR-19b (WT) and the GJA1 mRNA with mutated binding site of miR-19b (mutant) were cloned into the luciferase expressing pMIR vector respectively (Figure 3C). Each of the reconstructed reporter plasmids was transfected into HEK293T cells that were pretreated with miR-19b mimics or scrambled oligonucleotides controls (miR-NC). The results showed that miR-19b reduced the relative luciferase activity in cells transfected with GJA1-3’-UTR binding site, but not in cells transfected with mutated GJA1-3’-UTR (Figure 3D). These results verified that GJA1 was a direct target of miR-19b.

### 2.6. Re-Expression of GJA1 Rescued the miR-19b-Mediated Irregular Beating in hiPSCs-CMs

**Figure 4:**
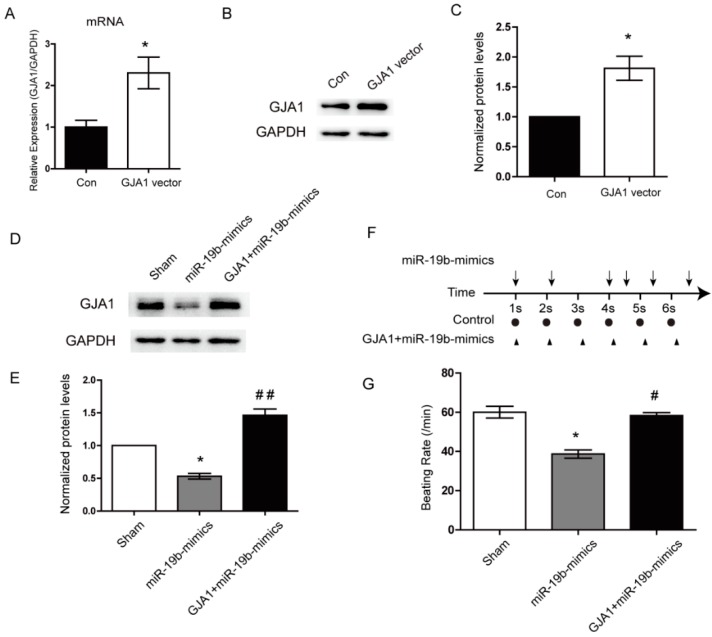
Overexpression of miR-19b led to irregular beating by decreasing the GJA1. (**A**) The transfection efficiency of GJA1 expression plasmid was assessed in hiPSCs-CMs by qPCR; (**B**,**C**) Western blot assay detected the transfection efficiency of GJA1 expression vector; (**D**,**E**) Western blot assay revealed that miR-19b mimics could significantly decrease the GJA1 protein level in hiPSC-CMs, while the GJA1 vector could restore the above effect; (**F**) The beating patterns of cardiomyocytes in different groups. Arrows represented the hiPSCs-CMs which were transfected with miR-19b mimics. The triangle represented the hiPSCs-CMs which were transfected with the GJA1 vector and miR-19b mimics; (**G**) The beating rate of hiPSCs-CMs was shown in different groups. The enhanced GJA1could rescue the beating rate. *n* = 3 per group. * *p* < 0.05 *vs*. control. ^#^ *p* < 0.05, ^##^ *p* < 0.01 *vs.* the group of miR-19b mimics.

To explore whether GJA1 could blunt the miR-19b-mediated irregular beating, we re-expressed the GJA1 in the hiPSCs-CMs using a specific overexpression plasmid. The transfection efficiency of this plasmid was verified using qPCR and western blotting (Figure 4A–C). Then the expression plasmid of GJA1 was transfected into the hiPSCs-CMs which had already been transfected with miR-19b mimics. As compared with the sham group, miR-19b mimics remarkably decreased the expression of GJA1, confirming the negative regulation of GJA1 by miR-19b. More importantly, re-expression of GJA1 significantly blunted the miR-19b-mediated downregulation of GJA1 in hiPSCs-CMs (Figure 4D,E). Furthermore, when GJA1 was downregulated by miR-19b mimics, hiPSCs-CMs beat irregularly which displayed an erratic rhythm and the beating rate was decreased. However, after re-expression of GJA1, hiPSCs-CMs maintained a nearly periodic beating pattern and the beating rate was also recovered to the normal level (Figure 4F,G). These results demonstrated that as the target of miR-19b, GJA1 could functionally rescue the miR-19b-mediated irregular beating in the hiPSCs-CMs. 

### 2.7. miR-19b Cooperated with miR-1 to Regulate the GJA1 Expression

**Figure 5:**
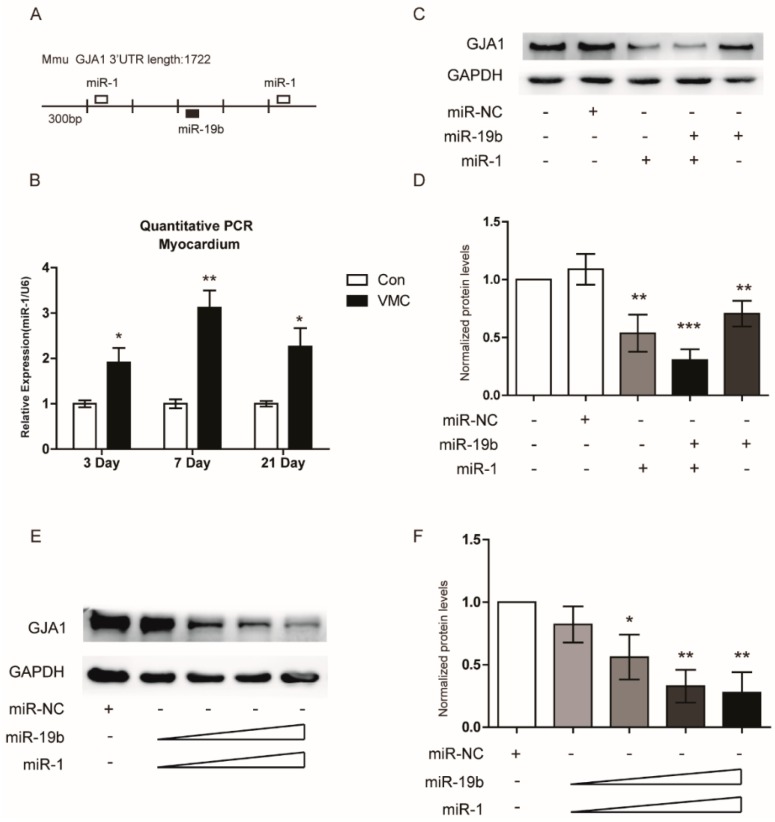
miR-19b and miR-1 cooperated to inhibit the GJA1 expression in HL-1 cells and hiPSC-CMs. (**A**) Bioinformatics analysis predicted the possible target sites of miR-19b and miR-1 in the 3’ UTR of GJA1; (**B**) qRT-PCR revealed miR-1 was increased at the indicated time of VMC mice model. *n* = 6 per group; (**C**,**D**) miR-19b and miR-1 mimics (50 nM) were transfected into HL-1 cells individually or in combination. The according changes of GJA1 levels were examined by western blot; (**E**,**F**) Various doses of miR-19b and miR-1 mimics (25, 50, 75, 100 nM) were cotransfected in combination into hiPSC-CMs. GJA1 protein levels were analyzed by western blot. *n* = 3 per group. * *p* < 0.05, ** *p* < 0.01 *** *p* < 0.001 *vs*. negative control.

Our previous study has found that miR-1 could suppress the GJA1 expression in VMC in the same way as miR-19b did [14]. Analysis of the 3’-UTR of GJA1 mRNA revealed that the two binding sites of miR-1 were adjacent to that of miR-19b (Figure 5A). MiR-1 shared a similar expression tendency with miR-19b during the progression of VMC (Figure 5B). Therefore, we assumed that miR-19b could interact with miR-1 in VMC. In order to prove this hypothesis, both miRNA mimics were transfected into HL-1 cells combinedly or separately. Notably, co-transfection of both miRNAs caused stronger inhibition of GJA1 expression than individual transfection (Figure 5C,D). To further confirm these results, we transfected these two miRNAs at different doses into the hiPSCs-CMs. Western blot showed that along with the increasing concentrations of both mimics, the GJA1 expression was dose-dependently decreased (Figure 5E,F). These consistent data obtained from HL-1 cells and hiPSCs-CMs strengthened the notion that miR-19b could cooperate with miR-1 to down-regulate the GJA1 expression.

## 3. Discussion

Viral myocarditis (VMC) is a frequent cause of sudden death by often triggering cardiac arrhythmia [18]. Several researches demonstrated that the descent expression of gap junction proteins which play important roles in communication between cardiomyoctyes could invoke arrhythmias [6,19,20,21]. GJA1, the major protein of gap junctions, is responsible for synchronous contraction by forming the cell-to-cell pathways for orderly spread of the wave of electrical excitation [21]. Reduced GJA1 can increase the propensity for arrhythmias by rendering the ventricle more susceptible to re-entry [22]. The reduction of about 90% in GJA1 expression can result in about 50% decrease of the conduction velocity [8]. As reported by Gutstein *et al*., cardiac deletion of GJA1 could result in spontaneous ventricular arrhythmias without left ventricle dysfunction, similar to what was seen in VMC [18,20]. These changes strongly implicated that alteration in myocardial expression of gap junctions is highly correlated with arrhythmogenesis [7]. Our current results found that with the development of VMC, the expression level of GJA1 was diminished by miR-19b gradually. And the loss of GJA1 could result in irregular beating pattern in the *in vitro* assay using hiPSCs-CMs. These findings were consistent with previous reports.

These hiPSCs-CMs are advantageous to the research of heart disease, especially for the study of cardiac rhythm. It is well known that human cardiac tissues are difficult to procure for research and cannot be cultured easily *in vitro*, therefore their utility for studying the mechanisms of cardiac diseases is limited [23]. However, hiPSCs-CMs can be abundantly produced *in vitro* [24]. Compared with the commonly used cardiac cell lines such as HL-1 or H9C2, the advantage of hiPSCs-CMs is that they are more structurally and physiologically simulative to the adult human cardiomyocytes. In comparison, HL-1 cells are an immortalized mouse cardiac cell line derived from a tumor lineage that does not reflect adult human cardiomyocytes [25]. As reported by Sinnecker *et al*., hiPSCs-CMs could provide a novel platform for studying the mechanisms of CVB_3_-induced viral myocarditis [26]. Hence, hiPSCs-CMs were utilized to study the functional role of miR-19b *in vitro*.

Accumulating studies have implicated miRNAs in the pathologenesis of VMC [27]. Our previous study also found that miR-1 was involved in VMC via post-transcriptional repression of GJA1 [14], and miR-21 regulated the progression of VMC to dilated cardiomyopathy (DCM) [28]. In the present study, miR-19b was highly expressed throughout the progression of VMC. MiR-19b is a member of the miR-17-92 cluster, which has been reported to mediate a variety of diseases [15]. In cardiac hypertrophy, the miR-19a/b family positively regulates cardiomyocytes hypertrophy by targeting atrogin-1 and MuRF-1 [29]. In addition, overexpression of miR-19b impacts left-right symmetry by targeting ctnnb1 in the cardiac development of zebrafish embryos [30]. In contrast to the upregulation of miR-19b in cardiac hypertrophy and cardiac development, miR-19b is downregulated in coronary artery disease, which is closely linked to the Apaf1/caspase-dependent pathway [31]. These reports have collectively suggested the great involvement of miR-19b in heart diseases. The present study revealed that overexpression of miR-19b could provoke irregular beating patterns in hiPSCs-CMs, indicating that miR-19b might be associated with arrhythmias. Moreover, the GJA1 which plays important roles in the maintenance of normal cardiac rhythm [7], was revealed to be negatively targeted by miR-19b using systemic *in vitro* assays. Our findings were in consistent with a previous study which showed overexpression of miR-19b could induce arrhythmias by targeting the GJA1 in transgenic mice [32]. More interestingly, re-expression of GJA1 functionally rescued the miR-19b-mediated irregular beating in hiPSCs-CMs. Based on previous studies and the present observation, it is conclusive that miR-19b may contribute to cardiac arrhythmia by repression of GJA1 in VMC.

Several recent studies have highlighted the role of miRNAs in the regulation of gap junction proteins and pointed toward a network of miRNAs for critical control of cardiac rhythm. For instance, miR-23a participates in estrogen deficiency-induced gap junction remodeling of rats by targeting GJA1 [33], while miR-130a was found to downregulate GJA1, resulting in cardiac arrhythmias [34]. Yang B *et al*. showed that miR-1 regulates cardiac arrhythmogenic potential by targeting GJA1 and KCNJ2 [10]. Our previous study also demonstrated that miR-1 was able to decrease the expression of GJA1 in VMC [14]. The present study found that miR-19b could cooperate with miR-1 to diminish the GJA1 expression in a dose-dependent manner. These researches indicated the regulation of GJA1 is not decided by one single miRNA, but by a network of miRNAs that contribute to the eventual reduction.

There are several limitations in this study. First, the pathogenesis of several types of cardiomyopathy including diabetic, hypertensive or hypertrophic, were shown to be controlled by profibrotic genes [35]. Based on our observation that there was significant fibrosis in late stage of VMC, we speculated that miR-19b might also regulate profibrotic genes other than the arrhythmogenic gene *GJA1* in VMC. Second, hiPSCs-CMs are ideal tools for cardiac arrhythmia in VMC [36]. But purity of the induced human cardiomyocytes limits their applications to a wider spectrum of diseases. Hence, it merits further investigation of how to produce hiPSCs-CMs with higher purity.

## 4. Materials and Methods

### 4.1. Animals

Male Balb/c mice (3–4 weeks old) were bred and maintained in the Institute of Laboratory Animal Sciences, Fudan University (Shanghai, China). All the protocols for the animal experiments were approved by the Institutional Animal Care and Use Committee of Fudan University. A Nancy strain of CVB_3_ was kindly provided by Ruizhen Chen from Zhongshan Hospital of Fudan University. Virus titer was determined by a 50% tissue culture infectious dose (TCID50) assay on HeLa cell monolayer and calculated by the Reed-Muench method. Mice were randomly divided into 3 groups which were subject to sacrifice on different time after inoculation (day 3, 7 and 21 respectively). Each group of mice was equally separated into 2 subgroups, the VMC group and the paired control group. The VMC group was intraperitoneally (i.p.) injected with 0.1 mL of Dulbecco’s Modified Eagle’s Medium (DMEM) containing 10^5^ TCID50 CVB_3_. The control group was mock-infected intraperitoneally with DMEM. The first day on inoculation was designated as day 0.

### 4.2. Histopathological Analysis and Myocardial Pathological Scoring

Hearts were harvested from infected mice on Day 3, 7, and 21 after i.p. injection, and were immediately fixed in 4% formalin solution, neutral buffered and then embedded in paraffin. Then the specimens were sectioned into 4 μm thick slides and stained with hematoxylin and eosin for histological diagnosis. The extent of myocardial lesions was quantified and pathological lesions were scored (Table 1) based on a previously established method with minor modifications [37]. The analysis was performed in a blinded manner by two trained pathologists.

### 4.3. Immunohistochemistry (IHC)

Sections of heart tissue were heated by a microwave in citrate buffer (pH = 6.0) and treated with 3% H_2_O_2_ for 15 min to abolish endogenous peroxidase activity. After blocked with 3% BSA-PBS for 30 min, sections were incubated with the primary rabbit anti-GJA1 antibody (Catalog No. 15386-1-AP, Proteintech Co., Chicago, IL, USA) at 4 °C overnight. A negative control using phosphate buffered saline instead of the specific primary antibody was synchronously set. Following incubation with goat anti-rabbit HRP-conjugated secondary antibody (Catalog No. sc-2004, Santa Cruz Biotech, Santa Cruz, CA, USA) for 30 min at 37 °C, the sections were incubated with 3,3-diaminobenzidine solution at room temperature for visualization. The tissue samples were subsequently dehydrated and mounted using neutral balsam.

### 4.4. Western Blot Analysis

Total proteins were extracted using RIPA buffer supplemented with serine protease inhibitor, phenylmethanesulfonylfluoride (PMSF). An equal amount of protein samples were resolved by 10% SDS-PAGE and transferred to PVDF membranes. Then the membranes were blocked with 5% non-fat dry milk in TBST for 1 h, followed by incubation with primary antibodies targeting GJA1 (Catalog No. 15386-1-AP, Proteintech Co., Chicago, IL, USA), or GAPDH (Catalog No. sc-365062, Santa Cruz Biotech) at 4 °C overnight. After 1 h incubation with the appropriate HRP-conjugated secondary antibody (Catalog No. sc-2004, Santa Cruz Biotech) at room temperature for 1 h, signals were detected using ECL reagents (Catalog No. 34096, Thermo Fisher Co, San Francisco, CA, USA). Signal intensities were quantified with the ImageJ v1.28 program (Department of Health and Human Services, National Institutes of Health (NIH)) and normalized to GAPDH expression. 

### 4.5. RNA Analysis

Total RNAs were isolated using TRIzol (Catalog No.15596-026, Invitrogen, Carlsbad, CA, USA) according to manufacturer’s instructions. RNA was reversely transcribed to cDNA using PrimeScript RT-PCR kit (Catalog No. RR014, Takara Clontech, Tokyo, Japan) and then used for quantitative real-time PCR using SYBR Premix Ex TaqII (Catalog No. RR820, Takara Clontech) and 7500 Fast Real-Time PCR Systems (Applied Biosystems, Foster, CA, USA). MicroRNA levels were detected using PrimeScript miRNA RT-PCR kit (Catalog No.638315, Takara Clontech) according to manufacturer’s protocol. U6 and GAPDH were used as internal controls. Primer sequences for quantitative real-time PCR are listed in Table 2 and Table 3.

### 4.6. miRNA Microarray Analysis

For microarray analysis, heart tissues from control and VMC-bearing mice were collected on day 7. Total RNAs were prepared using TRIzol and subjected to a quality check using the bioanalyzer (Agilent Technologies, Santa Clara, CA, USA). An 8 × 12 K miRNA microarray (Agilent Technologies, Santa Clara, CA, USA) was used to visualize the expression profiles of miRNAs. All procedures were carried out according to the manufacturer’s instructions. The expressed miRNA data were normalized using the quantile normalization method. The threshold used to screen up- or down-regulated miRNAs was a fold change (FC) of ≥1.5 and *p* value ≤0.05.

### 4.7. Luciferase Activity Assay

A wild type GJA1-3’-UTR gene containing miR-19b-binding site (GJA1-3’UTR-wt) and a mutated GJA1-3’-UTR gene with mutated miR-19b-binding site (GJA1-3’UTR-mut) were constructed and separately cloned into pMIR-REPORT miRNA expression reporter vector (Ambion, Inc., Foster, CA, USA). The HEK293T cells were plated into 24-well plates and transfected with empty vector, GJA1-3’-UTR-wt, GJA1-3’UTR-mut along with miR-19b mimic or scramble miR mimic. After 48 h of transfection, the cells were collected for fluorescence examination using Dual-Luciferase Reporter Assay System (Promega Co., Madison, WI, USA).

### 4.8. Cell Culture and Transfection in Vitro

The mouse atrial cardiomyocyte cell line, HL-1 (kindly provided by William C. Claycomb from Louisiana State University), was cultured in Claycomb’s growth medium. The human induced pluripotent stem cells derived cardiomyocytes (hiPSCs-CMs) which derived from the human skin fibroblasts were kindly provided by Ning Sun from School of Basic Medical Sciences, Fudan University and were cultured in DMEM supplemented with 5% FBS. The cardiac differentiation of the iPSCs is based on an optimized cardiac differentiation strategy using the chemically defined medium [38]. Transfection of each reagent into cells was performed using Lipofectamine^®^ 3000 (Catalog No. L3000-001, Invitrogen). Specially the method of GJA1 transfection was based on the reference [39], and the transfection efficiency was showed in Figure S1 Cells were harvested 48 h after transfection. The miR-19b mimics were chemically synthesized double-stranded RNAs. Viral infection of cells was conducted by incubation with viruses for 1 h in a serum-free medium followed by switching to complete medium. Cells were collected 24 h postinfection for protein and RNA extraction.

### 4.9. Statistics

All experiments were conducted independently at least three times. Data were presented as means ± SEM. Statistical significance was estimated with an ANOVA test and the Student’s *t* test using Prism (version 5, GraphPad Software, San Diego, CA, USA). *p* < 0.05 was considered statistically significant.

## 5. Conclusions

In all, our data suggest that miR-19b may contribute to cardiac arrhythmia through repression of GJA1 in VMC. And miR-19b could cooperate with miR-1 to diminish the GJA1 expression in a dose-dependent manner. Our data might give a new insight into the molecular mechanism of VMC.

## Figures and Tables

**Table 1:** The method for pathological scoring.

Score	Pathological Display
0	no inflammatory infiltrates
1	small foci of inflammatory cells between myocytes and the total inflammatory infiltrate area was less than 5% of the cross section
2	the total inflammatory infiltrate area was between 5% and 10%
3	inflammatory infiltrate area was between 10% and 25%
4	more than 25% inflammatory infiltrates or diffuse fibrosis lesions with necrosis

**Table 2:** Specific primers for real-time PCR analysis of miRNA.

miRNAs	Primer Sequences 5’-3’
mmu-miR-19b-3p	TGTGCAAATCCATGCAAAACTGA
mmu-miR-1a-3p	TGGAATGTAAAGAAGTATGTAT
U6 Forward	CAAGGATGACACGCAAATTCG
U6 Reverse	ACACGCAAATTCGTGAAGC

**Table 3:** Gene primers used for RT-PCR and real-time PCR.

Genes	Primer Sequences 5’-3’	
GJA1	Forward	CACTGAGCCCATCCAAAGA
	Reverse	TGTACCCAGGAGGAGACATAG
GAPDH	Forward	TGCGACTTCAACAGCAACTC
	Reverse	ATGTAGGCCATGAGGTCCAC

## Supplementary Materials

Supplementary materials can be found at http://www.mdpi.com/1422-0067/17/5/741/s1. Figure S1: Transfection efficiency of miR-19b and miR-1 in iPSCs-CMs and HL-1 cells. Video S1: The beating pattern of control hiPSCs-CMs. Video S2: The beating pattern of miR-19b mimics-treated hiPSCs-CMs.

## Abbreviations

VMC	Viral myocarditis
MiRNAs	MircroRNAs
GJA1	Gap junction protein alpha 1
hiPSCs-CMs	human cardiomyocytes derived from the induced pluripotent stem cells
CVB_3_	Coxsackievirus B_3_
3’-UTR	3’-untranslated region
TCID50	50% tissue culture infectious dose
i.p.	Intraperitoneally
DMEM	Dulbecco’s Modified Eagle’s Medium
