# Supplementary material for: MicroRNA-19b Downregulates Gap Junction Protein Alpha1 and Synergizes with MicroRNA-1 in Viral Myocarditis
Source: Int J Mol Sci. 2016 May 18;17(5):741. doi: 10.3390/ijms17050741 (PMC4881563; doi:10.3390/ijms17050741)
Supplement: Supplementary file 1 [file ijms-17-00741-s001.zip › ijms-126990-Supplementary Materials/Figure S1.pdf]

Supplementary Materials: MicroRNA-19b Downregulates Gap Junction Protein Alpha1 and Synergizes with MicroRNA-1 in Viral Myocarditis

Junyi Lin, Aimin Xue, Liliang Li, Beixu Li, Yuhua Li, Yiwen Shen, Ning Sun, Ruizhen Chen, Hongfei Xu and Ziqin Zhao

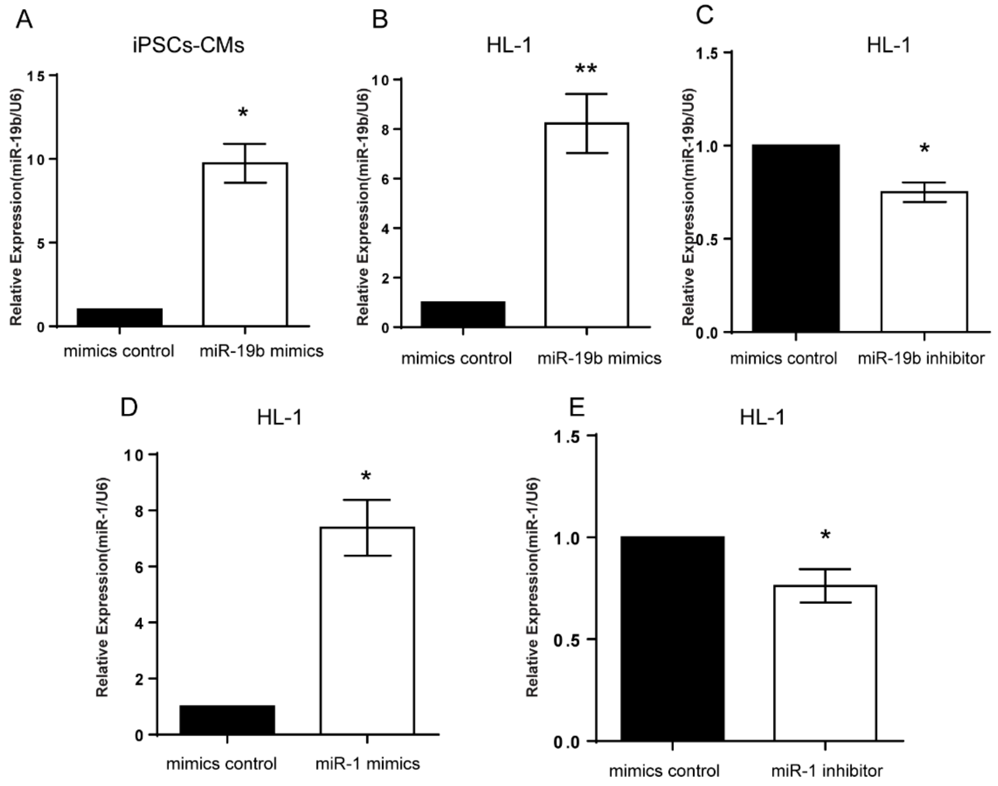

Figure S1. Transfection efficiency of miR-19b and miR-1 in iPSCs-CMs and HL-1 cells. (**A**) The transfection efficiency of miR-19b in iPSCs-CMs; (**B**,**C**) The transfection efficiency of miR-19b in HL-1; (**D**,**E**) The transfection efficiency. $n = 3$ per group. * $p < 0.05$, ** $p < 0.01$.
